# Impact of Statin Use on Immunotherapy Outcomes and Efficacy in Non-Small Cell Lung Cancer Patients

**DOI:** 10.3390/ijms27031541

**Published:** 2026-02-04

**Authors:** Alexander Yakobson, Abed Agbarya, Yulia Dudnik, Itamar Gothelf, Asmah Miari, Ronen Brenner, Ashraf Abu Jama, Nashat Abu Yasin, Abd El Nazer Dabah, Amichay Meirovitz, Natalie Maimon Rabinovich, Walid Shalata

**Affiliations:** 1 The Legacy Heritage Cancer Center, Dr. Larry Norton Institute, Soroka Medical Center, Beer Sheva 84105, Israel; 2 Faculty of Health Sciences, Ben Gurion University of the Negev, Beer Sheva 84105, Israel; 3 Department of Oncology, Bnai Zion Medical Center, Haifa 31048, Israel; 4 Goldman Medical School, Faculty of Health Sciences, Ben-Gurion University of the Negev, Beer-Sheva 84105, Israel; 5 Department of Oncology, Meir Medical Center, Kfar Saba 44180, Israel; 6 Faculty of Medicine, Tel Aviv University, Tel Aviv 69978, Israel; 7 Oncology Institute, Edith Wolfson Medical Center, Holon 58220, Israel

**Keywords:** statin, immunotherapy, non-small cell lung cancer, adenocarcinoma, squamous cell carcinoma, PD-L1, PD-1

## Abstract

Immune checkpoint inhibitors (ICIs) have improved outcomes in advanced non-small cell lung cancer (NSCLC). The influence of statin use, chemotherapy, PD-L1 expression, and sex on immunotherapy outcomes remains incompletely defined in real-world settings. We performed a multicenter retrospective analysis of patients with advanced NSCLC treated with immunotherapy-based regimens. Patients were stratified by statin exposure, chemotherapy use, PD-L1 expression (<1% vs. ≥1%), and sex. Overall survival (OS) and progression-free survival (PFS) were analyzed using Kaplan–Meier estimates and log-rank tests. Statin use was not associated with a significant OS benefit, while a numerical improvement in PFS was observed in selected subgroups. Among immunotherapy-treated patients, OS did not differ significantly by chemotherapy or statin use (median range, 19–27 months), whereas PFS differed significantly, with the longest PFS observed in patients receiving immunotherapy plus statins (26 months; *p* = 0.046). PD-L1 expression was the strongest determinant of outcomes, with PD-L1 ≥ 1% tumors demonstrating markedly longer OS and PFS compared with PD-L1 < 1% disease (OS up to 31 vs. 16 months; PFS up to 21 vs. 12 months; *p* < 0.001). No significant differences in OS or PFS were observed by sex or statin exposure (OS, 23–27 months; PFS, 14–19 months). In this real-world cohort, PD-L1 expression remained the primary predictor of survival outcomes following immunotherapy. Statin use was associated with modest PFS improvements but no consistent OS benefit, while sex did not significantly influence outcomes. These findings support continued reliance on established biomarkers and warrant prospective evaluation of statins as potential adjuncts to immunotherapy.

## 1. Introduction

Lung cancer is one of the most common types of cancer and a leading cause of cancer deaths worldwide. It occurs when abnormal cells grow uncontrollably in the tissues of the lungs, often forming tumors that interfere with respiratory function [1,2]. According to the World Health Organization, lung cancer remains the leading cause of cancer-related deaths globally, responsible for approximately 1.8 million deaths annually. The incidence of lung cancer varies significantly by region, with higher rates generally observed in countries with high tobacco use. Smoking remains the primary risk factor, contributing to over 80% of lung cancer cases, though environmental exposures, occupational hazards, and genetic predisposition also play roles [3,4,5,6,7].

Lung cancer is broadly categorized into two main types: non-small-cell lung cancer (NSCLC), including adenocarcinoma, squamous cell carcinoma, and large cell carcinoma, accounting for about 85% of all lung cancers; and small cell lung cancer, making up the remaining 15% [3,4,5,6].

Historically, the prognosis for advanced NSCLC has been poor, with limited treatment options primarily consisting of chemotherapy and radiation, which often yielded modest survival benefits and considerable toxicity [6,8,9,10,11]. However, the advent of immunotherapy has transformed the therapeutic landscape of NSCLC, offering a new era of hope and improved clinical outcomes for patients. Immunotherapy, particularly immune checkpoint inhibitors (ICIs), works by reactivating the body’s own immune system to recognize and attack cancer cells. These therapies target regulatory pathways such as programmed death-1 (PD-1), programmed death-ligand 1 (PD-L1), and cytotoxic T-lymphocyte-associated protein 4 (CTLA-4), which tumors exploit to evade immune detection. By blocking these pathways, ICIs restore the immune system’s ability to identify and destroy malignant cells. Clinical trials and real-world evidence have demonstrated that immunotherapy can significantly improve overall survival and progression-free survival in patients with NSCLC, especially those with high PD-L1 expression [8,9,10,11,12]. The introduction of ICIs like nivolumab plus ipilimumab, pembrolizumab, and atezolizumab has not only extended life expectancy in advanced-stage disease but also provided durable responses in a subset of patients who previously had limited options [9,10,11,12].

Dyslipidemia has been identified as a significant contributor to carcinogenesis, tumor invasion, and metastasis. Cancer cells are known to exhibit heightened lipid biosynthesis, a process that supports the metabolic demands of rapid cell proliferation and supplies cholesterol necessary for membrane formation and cellular stability. Considering this, there has been growing interest in the potential anti-cancer effects of lipid-lowering agents, particularly statins [13,14,15]. Supporting this, a large retrospective study involving 146,326 women in the United States found that statin use was associated with a significantly reduced risk of cancer-related mortality, with a hazard ratio (HR) of 0.78 (95% CI, 0.71–0.86) compared to non-users [16]. Similarly, a comprehensive 15-year observational study from Denmark, encompassing various cancer types, reported a 15% reduction in all-cause mortality among cancer patients who were statin users (95% CI, 13–17), further highlighting the potential therapeutic relevance of statins in oncology [17].

Immunotherapy is the standard of care for wild-type metastatic NSCLC [18]. While recent evidence suggests that statins may modulate the tumor microenvironment and influence ICI efficacy, clinical findings remain inconsistent. To address this gap, we conducted a retrospective cohort study to evaluate the association between concurrent statin use and clinical outcomes in patients with advanced NSCLC. This article examines the impact of statins on OS and PFS, further stratifying results by immunotherapy-based regimens, PD-L1 expression levels, gender, smoking status, ECOG, and pathological results to provide a more nuanced understanding of their therapeutic role.

## 2. Results

The study cohort comprised 391 patients with advanced NSCLC, of whom 141 (36.1%) were receiving statins and 250 (63.9%) were not. The median age of the overall population was 67.4 years (range, 41–87); patients in the statin group were significantly older than those not receiving statins (median 70.2 vs. 65.8 years, *p* = 0.021).

Overall, the cohort was predominantly male (69.3%), with no significant difference in sex distribution between statin users and non-users (*p* = 0.64). Adenocarcinoma was the most frequent histologic subtype (67.8%), followed by squamous cell carcinoma (32.2%), with comparable proportions observed between the statin and non-statin groups (*p* = 0.53).

Most patients had a history of smoking, with 47.6% being current smokers and 37.1% former smokers, while never-smokers accounted for 14.6% of the cohort. Smoking status did not differ significantly between statin users and non-users (*p* = 0.71).

At treatment initiation, ECOG performance status (PS) 0–1 was observed in 82.6% of patients, indicating a generally preserved functional status. Notably, patients receiving statins were more likely to have ECOG PS 0 compared with non-statin users (35.5% vs. 24.8%), whereas ECOG PS 1 was more frequent among patients not receiving statins (58.0% vs. 46.8%), resulting in a statistically significant difference in ECOG distribution between groups (*p* = 0.048). The proportion of patients with ECOG PS ≥ 2 was similar in both groups.

Regarding treatment strategy, the majority of patients received chemo-immunotherapy (79.3%), particularly among non-statin users (82.4% vs. 73.8%), whereas immunotherapy alone was more frequently administered in the statin group (26.2% vs. 17.6%), with this difference reaching statistical significance (*p* = 0.039). Pembrolizumab was the most commonly used immune checkpoint inhibitor (69.1%), followed by ipilimumab plus nivolumab (30.9%), with no significant difference in distribution between statin users and non-users (*p* = 0.46).

Assessment of PD-L1 expression demonstrated that 59.8% of patients had PD-L1 ≥ 1%, with a slightly higher prevalence among statin users compared with non-statin users (61.7% vs. 58.8%); however, this difference was not statistically significant (*p* = 0.58), (Table 1).

**Figure 1:**
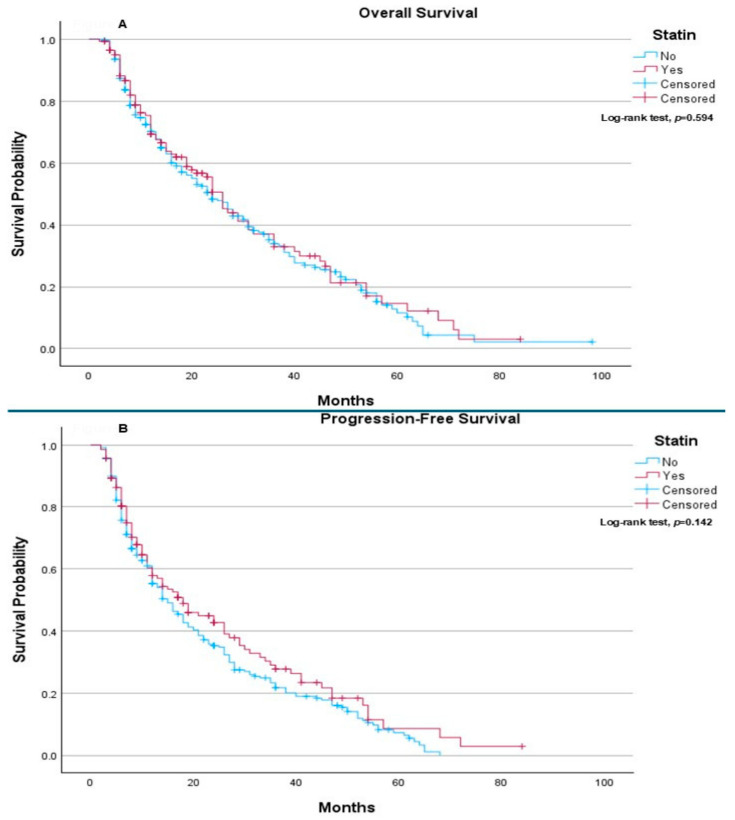
The use of statin vs. no statin as previous chronic treatment: (**A**) Overall Survival: The median overall survival was 24.0 months (95% CI: 19.59–28.41) for patients who did not take statins and 26.0 months (95% CI: 22.59–29.41) for patients who took statins. The log-rank test showed no statistically significant difference in overall survival between the groups (*p* = 0.594). (**B**) Progression-Free Survival: The median progression-free survival was 15.0 months (95% CI: 11.98–18.02) for patients who did not take statins and 18.0 months (95% CI: 12.13–23.87) for patients who took statins. The log-rank test showed no statistically significant difference between the groups (*p* = 0.142).

Regarding overall survival (OS), no statistically significant difference was observed between patients who received statins and those who did not (*p* = 0.594) (Figure 1A). The median OS was 24 months in the non-statin group and 26 months in the statin group. Similarly, progression-free survival (PFS) did not differ significantly between the two groups, with median PFS of 15 months among patients not receiving statins and 18 months among statin users (*p* = 0.142) (Figure 1B).

**Figure 2:**
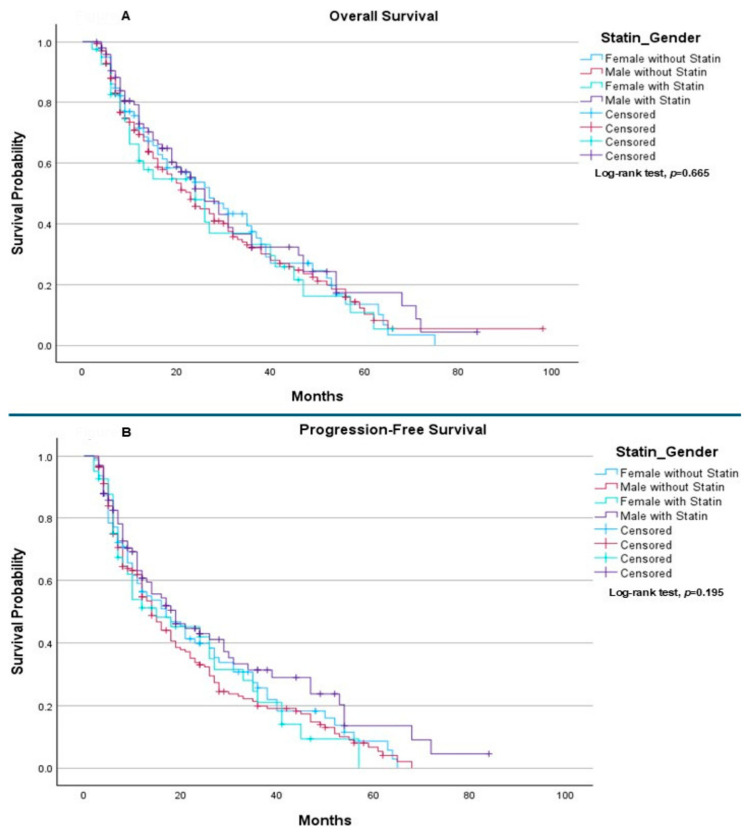
The effect of statin as chronic treatment on gender: (**A**) Overall Survival: The median overall survival was 27.0 months (95% CI: 19.11–34.89) for females without statin, 23.0 months (95% CI: 18.00–28.00) for males without statin, 24.0 months (95% CI: 11.47–36.53) for females with statin, and 26.0 months (95% CI: 20.10–31.90) for males with statin. The log-rank test showed no statistically significant difference in overall survival between the groups (*p* = 0.665). (**B**) Progression-Free Survival: The median progression-free survival was 17.0 months (95% CI: 10.48–23.52) for females without statin, 14.0 months (95% CI: 11.83–16.17) for males without statin, 15.0 months (95% CI: 3.06–26.95) for females with statin, and 19.0 months (95% CI: 12.18–25.82) for males with statin. The log-rank test showed no statistically significant difference between the groups (*p* = 0.195).

When analyses were stratified by gender, no statistically significant differences in survival outcomes were observed according to statin use. Median OS was 27.0 months for females without statins, 23.0 months for males without statins, 24.0 months for females receiving statins, and 26.0 months for males receiving statins (*p* = 0.665) (Figure 2A). Similarly, median PFS was 17.0 months for females without statins, 14.0 months for males without statins, 15.0 months for females receiving statins, and 19.0 months for males receiving statins, with no statistically significant differences observed (*p* = 0.195) (Figure 2B).

**Figure 3:**
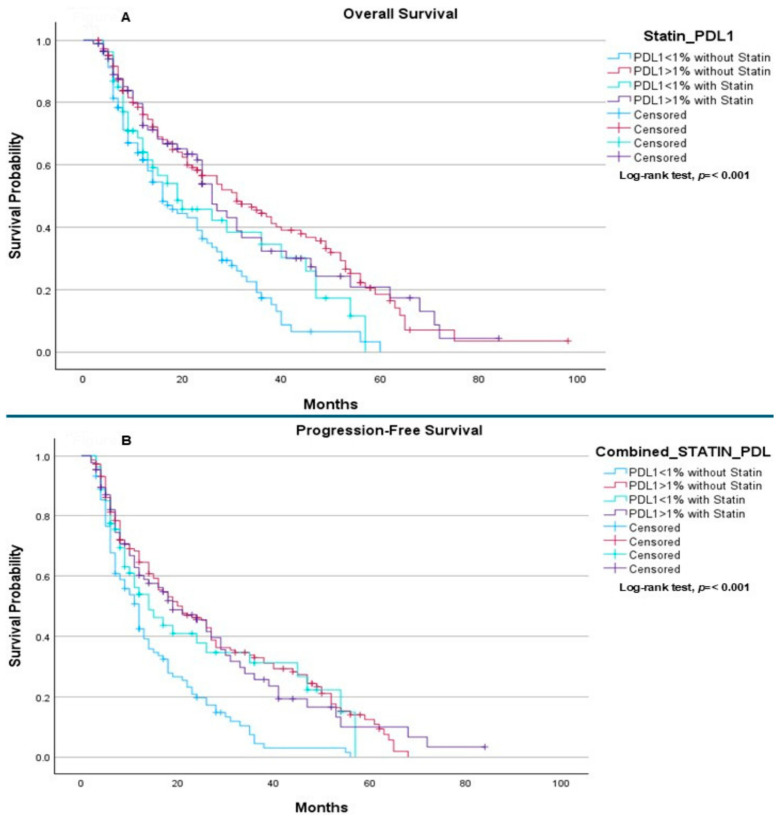
The correlations between the PD-L1 values and statin as chronic treatment: (**A**) Overall Survival: The median overall survival was 16.0 months (95% CI: 11.49–20.51) for patients with PD-L1 < 1% without statin, 31.0 months (95% CI: 22.21–39.79) for patients with PD-L1 > 1% without statin, 19.0 months (95% CI: 7.61–30.39) for patients with PD-L1 < 1% with statin, and 26.0 months (95% CI: 21.85–30.15) for patients with PD-L1 > 1% with statin. The log-rank test indicated a statistically significant difference in overall survival between the groups (*p* < 0.001). (**B**) Progression-Free Survival: The median progression-free survival was 12.0 months (95% CI: 9.82–14.18) for patients with PD-L1 < 1% without statin, 21.0 months (95% CI: 14.28–27.72) for patients with PD-L1 > 1% without statin, 14.0 months (95% CI: 7.14–20.86) for patients with PD-L1 < 1% with statin, and 19.0 months (95% CI: 11.30–26.70) for patients with PD-L1 > 1% with statin. The log-rank test indicated a statistically significant difference between the groups (*p* < 0.001).

When patients were stratified according to PD-L1 expression status (<1% vs. ≥1%) and statin use, a statistically significant difference in OS was observed across subgroups (*p* < 0.001). Median OS was 16.0 months for patients with PD-L1 < 1% not receiving statins, 31.0 months for those with PD-L1 ≥ 1% without statins, 19.0 months for patients with PD-L1 < 1% receiving statins, and 26.0 months for those with PD-L1 ≥ 1% receiving statins. The log-rank test confirmed a significant difference in OS among these groups (Figure 3A). A comparable pattern was observed for PFS, which also differed significantly across PD-L1 and statin subgroups (*p* < 0.001). Median PFS was 12.0 months in patients with PD-L1 < 1% without statins, 21.0 months in those with PD-L1 ≥ 1% without statins, 14.0 months in patients with PD-L1 < 1% receiving statins, and 19.0 months in those with PD-L1 ≥ 1% receiving statins (Figure 3B).

**Figure 4:**
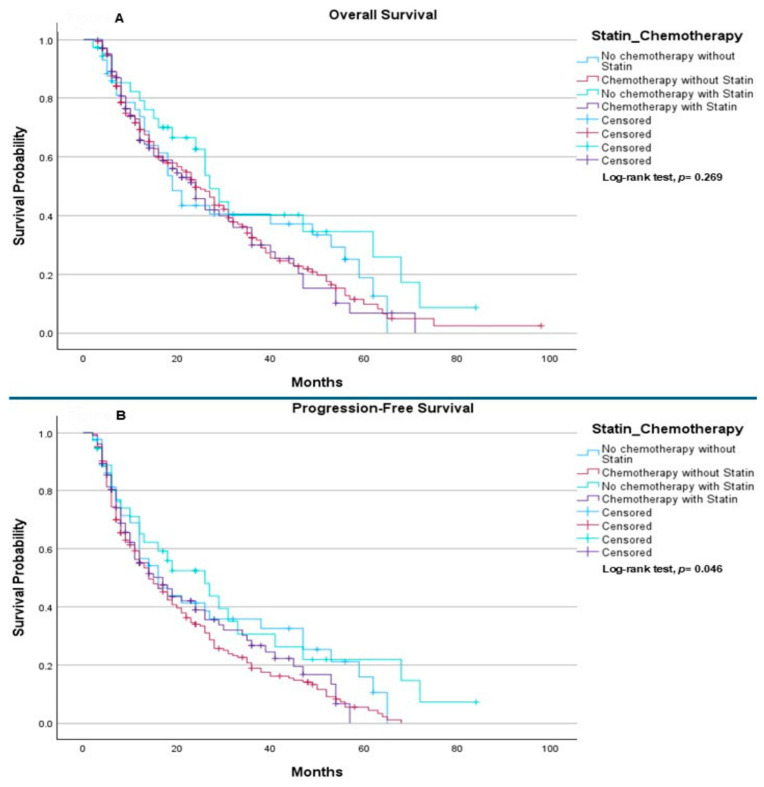
The correlations between type of treatment and statin as chronic treatment: (**A**) Overall Survival: The median OS was 19.0 months (95% CI: 14.68–23.32) for the Immunotherapy (IO) mono-therapy group; 24.0 months (95% CI: 19.63–28.37) for the Chemo-IO group; 27.0 months (95% CI: 21.70–32.30) for the Statin + IO group; and 24.0 months (95% CI: 18.31–29.69) for the Chemo-IO + Statin group. Log-rank testing demonstrated no statistically significant difference in OS across the four cohorts (*p* = 0.269). (**B**) Progression-Free Survival: The median PFS was 16.0 months (95% CI: 8.79–23.21) for the IO mono-therapy group; 14.0 months (95% CI: 10.68–17.32) for the Chemo-IO group; 26.0 months (95% CI: 13.00–39.00) for the Statin + IO group; and 17.0 months (95% CI: 10.79–23.21) for the Chemo-IO + Statin group. A statistically significant difference in PFS was observed between the groups (*p* = 0.046).

Finally, among patients treated with immunotherapy only and chemo-immunotherapy, the OS did not differ significantly between treatment subgroups (*p* = 0.269) (Figure 4A). Median OS was 19.0 months in patients receiving immunotherapy alone (without chemotherapy or statins), 24.0 months in those treated with chemo-immunotherapy, 27.0 months in patients receiving immunotherapy plus statins, and 24.0 months in those receiving both chemo-immunotherapy and statins. In contrast, PFS differed significantly across these subgroups (*p* = 0.046) (Figure 4B). Median PFS was 16.0 months for patients treated with immunotherapy alone, 14.0 months for those receiving chemo-immunotherapy, 26.0 months for patients receiving immunotherapy with statins, and 17.0 months for those receiving both chemo-immunotherapy and statins.

**Figure 5:**
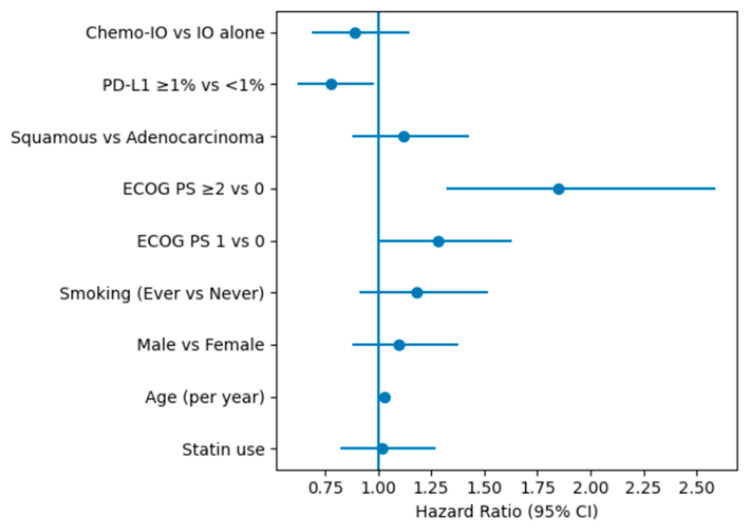
Forest plot of the multivariable Cox proportional hazards model for overall survival. The model included statin use, age, sex, smoking status, ECOG performance status, histology, PD-L1 expression, and treatment type. Increasing age and poorer ECOG performance status were independently associated with worse overall survival, whereas PD-L1 expression ≥ 1% was associated with improved survival. Statin use, sex, and smoking status were not independently associated with overall survival after multivariable adjustment.

To rigorously evaluate the impact of statin use, a multivariable Cox proportional hazards analysis was performed (Figure 5), adjusting for key covariates including age, sex, PDL-1 expression, smoking status, and ECOG performance status.

## 3. Discussion

Cancer has remained one of the most feared diseases since the 20th century and continues to show a rising incidence in the 21st century. It is also a leading cause of death worldwide. Recent data show that there were over 19 million new cancer cases in 2022 and nearly 10 million cancer-related deaths globally. Among these, lung cancer remained the primary cause of cancer-related mortality, accounting for approximately 1.8 million deaths (18%) [1,2,3,4].

Recently, the use of immunotherapy has brought a significant transformation in cancer treatment. It has not only proven effective in improving PFS, OS, and response rates but has also enhanced the quality of life for cancer patients compared to traditional chemotherapy, which has long been the standard of care [19,20,21]. Considering these advances, growing interest has emerged in understanding how immunotherapy outcomes can be further optimized, particularly in the context of patients receiving concurrent chronic treatments. One such commonly prescribed class of medications is statins. Statins are well-known for their cardiovascular benefits, including anti-inflammatory properties, reduced morbidity and mortality in hyperlipidemia, regression of coronary atherosclerosis, and a lowered risk of cerebrovascular events [13,14,22,23,24]. Their primary mechanism involves lipid-lowering through partial and reversible competitive inhibition of the enzyme HMG-CoA reductase, a key player in the mevalonate pathway involved in cholesterol synthesis. As a result, statins lead to decreased total and LDL (bad) cholesterol levels, increased HDL (good) cholesterol, and reduced triglyceride levels [14,24,25,26].

The rationale behind considering statins as potential enhancers of immunotherapy effectiveness stems from the role of dyslipidemia in cancer progression [13,14]. Dyslipidemia has been recognized as a key contributor to carcinogenesis, tumor invasion, and metastasis. Cancer cells often exhibit increased lipid biosynthesis to meet the metabolic demands of rapid proliferation and to supply the cholesterol required for membrane formation and cellular stability. Consequently, it is thought that the use of statins as a chronic treatment—especially when continued after a cancer diagnosis and during immunotherapy—may enhance therapeutic efficacy by disrupting lipid metabolism and supporting anti-tumor immune responses [13,14,15,16,17].

Moreover, statin use has been shown to play a potential role in cancer prevention. It has been associated with a reduced risk of developing various malignancies, including colorectal cancer—regardless of molecular subtype—as well as gastric cancer, hepatocellular carcinoma, and head and neck cancers [13,27,28,29,30]. Statins have also been linked to improved biochemical recurrence-free survival in patients undergoing curative treatment for prostate cancer [31,32]. In addition, several published studies, including systematic reviews and meta-analyses, have reported that statin use may improve OS and PFS in cancers such as gastric, prostate, and breast cancer [22,28,32,33,34,35,36,37,38]. Notably, the reduction in cancer-specific mortality was more pronounced in women with hormone receptor-positive/HER2-negative breast cancer [36,37,38], as well as in ovarian cancer [39]. However, the efficacy of statins in lung cancer—particularly NSCLC—remains uncertain and not well established. A previously published systematic review and meta-analysis of observational studies, which included 77 studies with a total of 98,445 patients, found that statin use had no significant impact on mortality or overall survival in this context [33,40].

However, these studies were conducted in the context of chemotherapy and tyrosine kinase inhibitor treatments, prior to the era of standard immunotherapy treatment.

Therefore, our study aimed to evaluate the impact of statin use on OS and PFS in patients receiving immunotherapy for lung cancer, with subgroup analyses based on various clinical and pathological factors.

Our findings revealed no statistically significant association between statin use and improvement in OS or PFS in the overall metastatic NSCLC population. This lack of effect may be influenced by geographical or environmental factors; however, it is consistent with previous evidence from systematic reviews and meta-analyses of observational studies, which also found that statin use did not significantly benefit the overall lung cancer population [33,40].

In this subgroup analysis, no statistically significant differences in OS were observed across the immunotherapy-based treatment cohorts, regardless of chemotherapy or statin use. While median OS was numerically higher in patients receiving statins—both as monotherapy and in combination with chemotherapy—these trends failed to reach statistical significance. This suggests that statin use may not independently drive long-term survival outcomes in this specific patient population.

In contrast, PFS differed significantly across treatment groups. Patients receiving immunotherapy in combination with statins demonstrated the longest median PFS, exceeding that observed in patients treated with immunotherapy alone or with chemo-immunotherapy. This finding raises the possibility that statins may enhance disease control or delay progression when used alongside immunotherapy. The lack of a similar improvement in PFS among patients receiving chemotherapy and statins may reflect underlying differences in disease burden, treatment sequencing, or patient selection. The discordance between PFS and OS outcomes may be explained by post-progression therapies, crossover effects, or the relatively limited follow-up duration, which could dilute potential survival benefits. Additionally, the retrospective nature of this analysis and potential confounding factors, such as comorbidities and indication bias for statin use, warrant cautious interpretation. Nevertheless, the observed association between statin use and improved PFS supports the hypothesis of a potential synergistic effect between statins and immune checkpoint inhibitors, which merits further investigation in prospective studies. These findings suggest a potential trend toward improved outcomes with statin use in the absence of chemotherapy.

In contrast, PD-L1 expression is a well-established biomarker and key determinant of response to immunotherapy, particularly in NSCLC, as was shown in previous clinical trials including Checkmate 227, Checkmate 9LA, Keynote 042, Keynote 189 and Keynote 407 [19,20,21,41,42]. Both OS and PFS were significantly associated with PD-L1 expression status, with clear quantitative differences observed across all subgroups. Patients with PD-L1 < 1% who did not receive statins had the poorest outcomes, with a median OS of 16.0 months and a median PFS of 12.0 months. In contrast, patients with PD-L1 ≥ 1% and no statin exposure experienced substantially improved survival, with median OS reaching 31.0 months and median PFS 21.0 months, underscoring the strong prognostic and predictive value of PD-L1 expression in the context of immunotherapy. Among patients receiving statins, survival outcomes were numerically improved within both PD-L1 strata, although the magnitude of benefit remained modest relative to the effect of PD-L1 status itself. In the PD-L1 < 1% subgroup, statin use was associated with an increase in median OS from 16.0 to 19.0 months and median PFS from 12.0 to 14.0 months, suggesting a potential incremental benefit in a population typically characterized by limited responsiveness to immune checkpoint inhibition. Similarly, in PD-L1 ≥ 1% patients, median OS was 26.0 months and median PFS 19.0 months among statin users, compared with 31.0 and 21.0 months, respectively, in those not receiving statins. Overall, these findings indicate that PD-L1 expression remains the dominant determinant of survival outcomes, with differences of up to 15 months in OS and 9 months in PFS observed between PD-L1–negative and PD-L1–positive tumors. While statin use was associated with small numerical improvements in survival—particularly in PD-L1–negative disease—it did not fully offset the prognostic disadvantage conferred by low PD-L1 expression. The statistically significant differences across groups (*p* < 0.001 for both OS and PFS) likely reflect the combined influence of tumor immunogenicity and treatment responsiveness, with any potential immunomodulatory effects of statins remaining secondary.

In this gender-based subgroup analysis, no statistically significant differences in OS or PFS were observed according to sex or statin use among patients treated with immunotherapy-based regimens. Median OS ranged from 23.0 to 27.0 months across the four subgroups, with females not receiving statins demonstrating the longest median OS (27.0 months), followed by males receiving statins (26.0 months). Similarly, median PFS ranged from 14.0 to 19.0 months, with numerically longer PFS observed in males receiving statins (19.0 months). However, these differences did not reach statistical significance, suggesting that neither sex nor statin exposure independently influenced survival outcomes in this cohort.

These findings are broadly consistent with results from pivotal phase III immunotherapy trials in advanced NSCLC. In CheckMate 227, nivolumab-based regimens demonstrated durable OS benefit compared with chemotherapy across both sexes, without evidence of clinically meaningful sex-related heterogeneity in treatment effect [19]. Likewise, CheckMate 9LA showed improved survival with nivolumab plus ipilimumab and short-course chemotherapy regardless of sex, supporting the notion that sex alone is not a dominant modifier of immunotherapy efficacy [20].

Similar conclusions can be drawn from pembrolizumab-based trials. In KEYNOTE-042, pembrolizumab monotherapy improved OS compared with chemotherapy in patients with PD-L1–expressing tumors, with benefit observed in both male and female patients [21]. KEYNOTE-189 and KEYNOTE-407 further established the survival advantage of chemo-immunotherapy combinations in non-squamous and squamous NSCLC, respectively, again without consistent sex-based differences in OS or PFS. The median survival outcomes observed in our real-world cohort—OS of approximately 23–27 months and PFS of 14–19 months—are comparable to those reported in these landmark studies, supporting the external validity of our findings [41,42].

Although preclinical and translational studies have suggested potential sex-related differences in immune response and tumor immunogenicity, the lack of statistically significant survival differences in our analysis aligns with the clinical trial evidence indicating that sex does not meaningfully alter outcomes with immune checkpoint inhibitors in NSCLC. Furthermore, while statins have been hypothesized to modulate antitumor immunity through effects on cholesterol metabolism and T-cell function, their use did not confer a consistent survival advantage in either females or males in this analysis.

Taken together, these results suggest that immunotherapy efficacy in advanced NSCLC is largely independent of patient sex and statin exposure, and that treatment decisions should continue to be guided primarily by established predictive factors such as PD-L1 expression, disease histology, and clinical fitness.

## 4. Materials and Methods

### 4.1. Patient Enrolment

This retrospective, observational, and non-interventional study focused on patients diagnosed with metastatic NSCLC (Soroka Medical Center), 236 patients (Bnai Zion Medical Center), 38 patients (Meir Medical Center 106) and 11 patients (Wolfson Medical Center). The analysis included individuals who were chronically treated with statins as chronic medication before diagnosis (at least 2 years) or not, and who received first-line chemo-immunotherapy according to standard clinical practice. Treatments consisted of either pembrolizumab or a combination of ipilimumab and nivolumab, administered with or without chemotherapy (as accepted for first line of therapy). The study period spanned from January 2018 to October 2024, with follow-up data collected up to November 2024. Information gathered included patient demographics (such as sex and age at diagnosis), treatment details (including whether patients received pembrolizumab or the ipilimumab-nivolumab regimen, with or without chemotherapy), and clinical outcomes such as OS (time from starting oncological treatment until death) and PFS (time from starting oncological treatment until disease progression). Additional variables documented were PD-L1 expression levels, treatment initiation and completion dates, progression sites, and Eastern Cooperative Oncology Group (ECOG) performance status scores.

### 4.2. Inclusion and Exclusion Criteria of the Study Population

#### 4.2.1. Inclusion Criteria

Patients were eligible for inclusion if they were 18 years of age or older and had a histologically confirmed diagnosis of NSCLC. Only those with confirmed negative status for EGFR, RET, ALK, BRAF, MET, and ROS mutations were included, in line with the standard first-line treatment protocols at the time. Eligible patients must have initiated therapy with immune checkpoint inhibitors, including pembrolizumab or the combination of ipilimumab and nivolumab, administered either as monotherapy or in combination with chemotherapy. To ensure adequate follow-up and treatment consistency, all patients must have received their care at the participating medical center, with complete inpatient records available for review. Medical records needed to contain comprehensive documentation of each patient’s underlying health conditions and chronic medication use, enabling clear identification of those receiving statin therapy and those who were not. Additionally, patients were required to have achieved PFS of more than four months during treatment. Given the retrospective nature of the study, patients with ECOG performance statuses ranging from 0 to 4 were deemed eligible and included.

#### 4.2.2. Exclusion Criteria

Patients were excluded from the study if their medical records were incomplete or lacked essential information regarding their medication history. Additionally, individuals who had received any form of systemic therapy for advanced or metastatic disease prior to the study were not eligible. This included those who had undergone immunotherapy or any other type of systemic treatment for any type of cancer at any point in time. These criteria were applied to minimize potential confounding factors and ensure that the study outcomes reflected the effects of first-line treatment only.

### 4.3. Treatment Administration and Doses

All patients underwent evaluation by a multidisciplinary team comprising pulmonologist, thoracic surgeon, pathologist, medical oncologist, radiation oncologist, nuclear medicine specialist, and radiologist. Clinical decisions were made based on pathological findings, imaging results, and the patient’s performance status. Each patient was assigned a primary physician responsible for overseeing his/her treatment plan. For those with locally advanced or metastatic NSCLC (any T stage, N1–3, and/or M1), a medical oncologist led the treatment in accordance with the National Comprehensive Cancer Network guidelines [18].

#### 4.3.1. Patients with Squamous Cell Carcinoma

For patients with squamous cell carcinoma, initial therapy included cisplatin or carboplatin (AUC 4–6) combined with paclitaxel (175 mg/m^2^) for the first two cycles. This was followed by maintenance with nivolumab and ipilimumab in accordance with the Checkmate 9LA protocol [19]. Alternatively, pembrolizumab (200 mg every three weeks) was administered following the four-cycle induction phase, according to the Keynote 407 regimen [20].

#### 4.3.2. Patients with Non-Squamous Histology

For patients with non-squamous histology, treatment was administered in three-week cycles, starting with intravenous cisplatin (75 mg/m^2^) or carboplatin (AUC 4–6) combined with pemetrexed (500 mg/m^2^). Immunotherapy agents nivolumab (360 mg every three weeks) and ipilimumab (1 mg/kg every six weeks) were included. After two cycles, the platinum-based agents were discontinued, and maintenance therapy with pemetrexed, nivolumab, and ipilimumab was continued, consistent with the Checkmate 9LA protocol [19]. Alternatively, pembrolizumab (200 mg every three weeks) was introduced after four initial chemotherapy cycles, following the Keynote 189 regimen [21]. All treatments were planned to continue for a maximum duration of two years or until disease progression or the occurrence of unacceptable toxicity. Patients receiving pemetrexed were premedicated with folic acid, vitamin B12, and glucocorticoids.

### 4.4. Statistical Analysis

Descriptive statistics were used to summarize the baseline demographic, clinical, and molecular characteristics of the study cohort. Continuous variables not following a normal distribution were presented as medians with ranges, while categorical variables were reported as frequencies and percentages. Kaplan–Meier survival analyses were employed to evaluate OS and PFS, comparing outcomes between patients with a history of statin use and those without. These analyses were further stratified based on key clinical variables, including PD-L1 expression levels, type of ICI administered, and histological subtype of NSCLC. The log-rank test was used to determine the statistical significance of differences observed in survival curves. To adjust for potential confounders, multivariable Cox proportional hazards regression models were applied to assess associations with both PFS and OS, yielding hazard ratios (HRs) with corresponding 95% confidence intervals (CIs). A *p*-value of less than 0.05 was considered statistically significant for all two-sided tests. All statistical analyses were conducted using SPSS software, version 29.0.

## 5. Conclusions

This study evaluated the impact of statin use on OS and PFS in patients receiving immunotherapy for lung cancer, with analyses across multiple clinical and pathological subgroups. Overall, statin use was not associated with statistically significant improvements in survival outcomes across the entire cohort or within subgroups, including those defined by gender, type of treatment immunotherapy alone or chemo-immunotherapy. While statins did not demonstrate a clear survival benefit on their own, certain subgroups, particularly those with high PD-L1 expressions showed trends that warrant further investigation. These findings highlight the importance of considering individual clinical and molecular factors when evaluating adjunctive therapies like statins in the context of immunotherapy.

## Figures and Tables

**Table 1:** Demographics of patient participants in our cohort, n = 391.

Characteristics	Overall (%), *N* = 391	Statin (%), *N* = 141 (36)	No Statin (%), *N* = 250 (64)	*p* Value
*Age (years)*				0.021
Median (range)	67.4 (41–87)	70.2 (52–82)	65.8 (41–68)	
*Gender, n (%)*				0.64
Female	120 (30.7)	41 (29.1)	79 (31.6)	
Male	271 (69.3)	100 (70.9)	171 (68.4)	
*Histology, n (%)*				0.53
Adenocarcinoma	265 (67.8)	93 (66)	172 (68.8)	
Squamous cell carcinoma	126 (32.2)	48 (34)	78 (31.2)	
*Smoking status, n (%)*				0.71
Never	57 (14.6)	18 (12.8)	39 (15.6)	
Current	186 (47.6)	70 (49.6)	116 (46.4)	
Past	145 (37.1)	51 (36.2)	94 (37.6)	
Unknown	3 (0.8)	2 (1.4)	1 (0.4)	
*ECOG, n (%)*				0.048
0	112 (28.6)	50 (35.5)	62 (24.8)	
1	211 (54.0)	66 (46.8)	145 (58)	
2+	68 (17.4)	25 (17.7)	43 (17.2)	
*Type of Treatment, n (%)*				0.039
Chemo-immunotherapy	310 (79.3)	104 (73.8)	206 (82.4)	
Only immunotherapy	81 (20.7)	37 (26.2)	44 (17.6)	
*Type of Immunotherapy, n (%)*				0.46
Pembrolizumab	270 (69.1)	94 (66.7)	176 (70.4)	
Ipilimumab plus Nivolumab	121 (30.9)	47 (33.3)	74 (29.6)	
*PD-L1 values n (%)*				0.58
*PD-L1* < 1%	157 (40.2)	54 (38.3)	103 (41.2)	
*PD-L1* ≥ 1%	234 (59.8)	87 (61.7)	147 (58.8)	

Abbreviation: N, number; ECOG, Eastern Cooperative Oncology Group.

## Data Availability

The original contributions presented in this study are included in the article. Further inquiries can be directed to the corresponding author.

## Abbreviations

The following abbreviations are used in this manuscript:

ICIs	Immune checkpoint inhibitors
NSCLC	Non-small cell lung cancer
PD-1	Programmed death-1
PD-L1	Programmed death-ligand-1
CTLA-4	Cytotoxic T-lymphocyte-associated protein 4
HR	Hazard ratio
SCC	Squamous cell carcinoma
ECOG	Eastern Cooperative Oncology Group
N	Number
OS	Overall survival
PFS	Progression-free survival
CIs	Confidence intervals
